# Understanding the Impact of Home Confinement on Children and Young People with ADHD and ASD During the COVID-19 Pandemic

**DOI:** 10.1007/s10578-022-01490-w

**Published:** 2023-01-12

**Authors:** Charlotte L. Hall, Christopher Partlett, Althea Z. Valentine, Samantha Pearcey, Kapil Sayal

**Affiliations:** 1 NIHR MindTech MedTech Co-operative, Institute of Mental Health, School of Medicine, Mental Health & Clinical Neurosciences, University of Nottingham, Innovation Park, Triumph Road, Nottingham, UK; 2 NIHR Nottingham Biomedical Research Centre, Institute of Mental Health, Mental Health & Clinical Neurosciences, University of Nottingham, Innovation Park, Triumph Road, Nottingham, UK; 3 Nottingham Clinical Trials Unit, School of Medicine, Applied Health Research Building, University Park, Nottingham, UK; 4 Department of Psychiatry, University of Oxford, Oxford, UK; 5 Department of Experimental Psychology, University of Oxford, Oxford, UK; 6 Institute of Mental Health, School of Medicine, Mental Health & Clinical Neurosciences, University of Nottingham, Innovation Park, Triumph Road, Nottingham, UK

**Keywords:** COVID-19, Mental health, Children and young people, ADHD, ASD

## Abstract

To understand whether the mental health of children and young people (CYP) with and without attention-deficit/hyperactivity disorder (ADHD) and/or autism spectrum disorder (ASD) were differentially affected by COVID-19. We analysed data (n = 6507) from the Co-Space study, a UK web-based longitudinal survey. CYP with ADHD (n = 160;2.5%), ASD (n = 465;7%), and ADHD + ASD (n = 155;2.4%) were compared with a reference group (n = 5727;88%) using parent-completed questionnaires [Strengths and Difficulties Questionnaire (SDQ) & Pandemic Anxiety Scale (PAS)]. Baseline to 1-month follow-up differences were compared using linear regression models. CYP with ADHD and/or ASD had higher scores at baseline than other CYP. At follow-up, CYP with ASD showed small but significant improvements in symptoms (SDQ), compared with the reference group. CYP with ASD experienced a worsening of disease anxiety (PAS) and CYP with ADHD a deterioration in functional impairment. These findings indicate a mixed pattern of pandemic-related impact for CYP with ADHD and/or ASD.

## Introduction

To control and minimise the rapid transmission of the Coronavirus disease 2019 (COVID-19), the United Kingdom (UK) government implemented lockdown restrictions, including home confinement and school closures at the end of March 2020. Consequently, the COVID-19 outbreak involved a rapid shift in social rules (e.g. rules surrounding personal space), as well as major changes to daily routines. Initial findings suggested that lockdown was associated with poor mental health for children and young people (CYP) [1, 2].

The UK National Health Service (NHS) highlighted CYP with neurodevelopmental disorders [NDDs such as attention-deficit/hyperactivity disorder (ADHD) and autism spectrum disorder (ASD)] as being particularly vulnerable to the negative impact of COVID-19 [3]. ADHD and ASD are estimated to affect around 3–5% and 1% of CYP respectively [5, 6]. Whereas ADHD is defined by impairing levels of inattention, impulsivity and hyperactivity, ASD is characterised by significant social communication difficulties and restrictive-repetitive behaviours [7]. There is considerable overlap between the disorders, the two are highly comorbid [6, 7] and both groups are at increased risk of depression and anxiety [8, 9]. Changes to routine can be a significant trigger for emotional and psychological distress for CYP with ADHD and/or ASD [4]. Whereas some CYP with ADHD/ASD may experience loneliness and increased risk of developing mental health problems in mainstream schools [10], schools provide structure, routine and facilitate contact with peers.

Cross-sectional studies and reports have highlighted the impact of COVID-19 on families living with ADHD and ASD. Pandemic mitigation responses such as home confinement or lockdown are reported to have contributed to an exacerbation of conduct problems and externalizing behaviours, increased irritability, and low mood for CYP with ADHD [11, 12] and post-traumatic stress disorder (PTSD) symptoms including aggression, hypersensitivity and behavioural difficulties, for CYP with ASD [13]. Parents have reported difficulties in managing hyperactivity due to quarantine restrictions limiting access to outdoors [11]. Disruption to therapeutic support or ongoing care services for families living with ASD have been noted, contributing to worsening ASD symptoms and family distress [14]. However, there is a mixed picture [15].The impact of lockdown is likely to have had a differential impact across different demographic groups and families from lower socio-economic groups may be particularly disadvantaged [15, 16]. For example, a recent review highlighted that physical exercise, access to entertainment, positive familial relationships, and social support were associated with better mental health outcome for CYP [17]. Similarly, families of CYP with NDDs have reported mixed impact. Although most parents of CYP with ASD have reported feeling overwhelmed, worrying about loss and changes in mood [18], some families have either reported improvements or no impact on mental health [19]. Comparably, one survey conducted at the start of lockdown about CYP with ADHD revealed that about one-third were doing better, one-third worse, and the remainder the same in terms of their ADHD, with factors such as sufficient space at home mediating outcomes [20]. Family demographics have been shown to mediate the effects of lockdown, with some CYP more likely to socialise, complete household chores and engage in personal care management than prior to the pandemic [13]. Individual and parental factors such as family cohesion have also been found to mediate levels of loneliness among CYP with ADHD [21]. However, longitudinal studies are scarce. A US study (n = 238) found adolescents with ADHD were more likely to experience worsening in inattention, hyperactivity/impulsivity and oppositional behaviours compared to adolescents without ADHD. This was further exacerbated when the adolescent had poor pre-pandemic emotion regulation [22]. There also appeared to be some socio-economic moderators whereby lower family income was associated with increased inattention but higher family come with increased oppositional/defiant behaviours. Similarly, a UK study (n = 527) found that CYP with ASD experienced more depression and anxiety symptoms than CYP with other special educational needs and disabilities. This depression and anxiety was also associated with belonging to a lower income household [23]. This study also showed that some CYP reported improved wellbeing from not having to attend school [24]. In contrast, a longitudinal Australian study (n = 476) showed little change in mental health symptoms for CYP with NDD pre/during the pandemic, but more positive wellbeing as a result of school closures [10]. Therefore, the effects on the pandemic for CYP with ADHD and ASD are not clear-cut and a clearer understanding is needed about the impact of the pandemic over time for this population so that support can be appropriately targeted and offered.

Although there is a growing body of literature on the impact of home confinement for CYP with ADHD and ASD, the research in Europe remains limited. In a cross-sectional study, using the Strengths and Difficulties Questionnaire (SDQ) at the start of the UK pandemic lockdown 371 CYP with ASD and ADHD were compared with data from previously published norms and a UK mental health cohort. Compared with neurotypical controls, CYP with NDD were more likely to show emotional symptoms (15%/42%), conduct problems (9%/28%), and fewer prosocial behaviours (22%/54%). Children and young people with ADHD showed greater conduct problems and CYP with ASD showed fewer prosocial behaviours than neurotypical CYP [25]. This study did not explore the role of contextual factors (such as home environment) on outcome. Importantly, this study also did not compare with neurotypical CYP at the same time point, and although the analyses adjusted for age, sex, and developmental level, the study did not account for the effects of consequences of lockdown which may act as additional risk factors for CYP with NDD. In particular, reduced access to outdoor space and physical activity [26] and reduced access to peers may be important exacerbating factors for these groups.

The Co-Space project [27] has been conducting an online longitudinal survey of parents and young people in the UK to understand the ongoing mental health impact of the COVID-19 pandemic. Using data obtained from the Co-Space project, the aim of this large longitudinal study is to investigate and understand the differences in mental health (including pandemic anxiety) for children and young people with ADHD and/or ASD compared with other CYP who did not have ADHD and/or ASD. This was achieved through comparing differences in the magnitude of changes in mental health and pandemic anxiety across these different diagnostic groups over a 1-month follow-up period. By better understanding the potential early impact from the pandemic and population-level mitigation approaches. This work is crucial in informing clinicians, practitioners, school pastoral staff and parents about appropriate care for this vulnerable population.

## Methods

### Participants

The participants in this study were 6507 parents and carers over the age of 18 years, reporting on one child aged between 4 and 16 years of age (Table 1). All participants were recruited as part of the Co-SPACE study (COVID-19: Supporting Parents, Adolescents and Children in an Epidemic; OSF protocol https://osf.io/8zx2y/). The Co-SPACE study gained ethical approval through the University of Oxford Medical Sciences Division Ethics Committee (reference R69060), and full details of these ethical considerations can be found in the OSF protocol. Participants completed a baseline survey and a follow-up, approximately 1 month apart (data collected between 30th March and 31st May 2020). All data were collected within the first UK lockdown, which saw the most stringent social restrictions between 23rd March 2020 and 1st June 2020. Parents reported on whether their child had any medical conditions including autism spectrum disorder (ASD), attention-deficit disorder or attention-deficit hyperactivity disorder (ADD/ADHD), clinically diagnosed anxiety or depression, or another clinically diagnosed mental health condition. Other UK research suggests that parent-reported diagnostic rates of ASD and ADHD approximate to clinical administrative prevalence [28]. For the purposes of this paper, CYP were categorised into the following diagnostic groups: (1) ADHD but no ASD, (2) ASD but no ADHD, (3) both ADHD and ASD, and (4) no or other mental health conditions (i.e. CYP who do not have ADHD or ASD). In this study, we use the term ‘parent’ to encompass both parents and legal guardians.

**Table 1:** Sample characteristics at baseline (n = 6507)

	ADHD and without ASD (n = 160)	ASD and without ADHD (n = 465)	ADHD and ASD (n = 155)	Reference group (n = 5727)	Total (n = 6507)
Child age					
Mean [sd]	10.4 [3.1]	10.6 [3.4]	10.3 [3.1]	9.3 [3.5]	9.4 [3.5]
Child gender					
Male	117 (73%)	294 (63%)	119 (77%)	2802 (49%)	3332 (51%)
Female	43 (27%)	157 (34%)	34 (22%)	2895 (51%)	3129 (48%)
Other/prefer not to say	0	14 (3%)	2 (1%)	30 (1%)	46 (1%)
Child ethnicity					
White: British, Irish, other	146 (91%)	431 (93%)	137 (88%)	5170 (90%)	5884 (90%)
Mixed race: White/Black & mixed race other	8 (5%)	21 (5%)	14 (9%)	303 (5%)	346 (5%)
Asian/British	1 (1%)	5 (1%)	0	100 (2%)	106 (2%)
Black/British	0	2 (< 1%)	1 (1%)	27 (< 1%)	30 (< 1%)
Other/prefer not to say	5 (3%)	6 (1%)	3 (2%)	127 (2%)	141 (2%)
Relationship of parent/guardian to child					
Parent	150 (94%)	457 (98%)	151 (97%)	5592 (98%)	6350 (98%)
Step-parent	2 (1%)	0	0	44 (1%)	46 (1%)
Grandparent	6 (4%)	4 (1%)	3 (2%)	38 (1%)	51 (1%)
Other	2 (1%)	4 (1%)	1 (1%)	53 (1%)	60 (1%)
Gender of parent/guardian					
Male	6 (4%)	19 (4%)	7 (5%)	424 (7%)	456 (7%)
Female	154 (96%)	443 (95%)	147 (95%)	5273 (92%)	6017 (92%)
Other/prefer not to say	0	3 (1%)	1 (1%)	30 (1%)	34 (1%)
Ethnicity of parent/guardian					
White: British, Irish, other	150 (94%)	445 (96%)	144 (93%)	5345 (93%)	6084 (93%)
Mixed race: White/Black & mixed race other	3 (2%)	7 (2%)	7 (5%)	113 (2%)	130 (2%)
Asian/British	2 (1%)	4 (1%)	0	113 (2%)	119 (2%)
Black/British		3 (1%)	1 (1%)	28 (< 1%)	32 (< 1%)
Other/prefer not to say	5 (3%)	6 (1%)	3 (2%)	128 (2%)	142 (2%)
Employment status of parent/guardian					
In education	3 (2%)	9 (2%)	3 (2%)	114 (2%)	129 (2%)
Employed	128 (80%)	317 (68%)	84 (54%)	4934 (86%)	5463 (84%)
Unable due to disability	9 (6%)	35 (8%)	21 (14%)	92 (2%)	157 (2%)
Homemaker/full-time parent	17 (11%)	98 (21%)	46 (30%)	491 (9%)	652 (10%)
Unemployed and seeking work	2 (1%)	3 (1%)	1 (1%)	75 (1%)	81 (1%)
Retired	1 (1%)	3 (1%)	0	21 (< 1%)	25 (< 1%)
Child has special education needs					
No	31 (19%)	34 (7%)	3 (2%)	5328 (93%)	5396 (83%)
Yes	129 (81%)	431 (93%)	152 (98%)	399 (7%)	1111 (17%)
Access to outside space					
No	7 (4%)	28 (6%)	9 (6%)	232 (4%)	276 (4%)
Yes	153 (96%)	437 (94%)	146 (94%)	5491 (96%)^a^	6227 (96%)^a^

Reference group contained children and young people with no or other mental health disorders that were not ADHD and/or ASD.

*ADHD* attention deficit hyperactivity disorder. *ASD* autism spectrum disorder.

^a^4 participants with missing data.

### Measures

*Strengths and Difficulties Questionnaire* (SDQ) was completed to assess the CYP’s mental health [29]. It consists of 25-items rated on a 3-point Likert scale (not/somewhat/certainly true), with higher scores indicating greater problem severity. There are five sub-scales: Emotional Symptoms, Conduct Problems, Hyperactivity/Inattention, Peer Problems and Pro-Social Behaviour. A total difficulties score is generated from the sum of the first four sub-scales. Associated functional impairment is assessed in terms of distress, and impact on home life, friendships, learning and leisure activities [30]. For the purpose of this analysis, results from emotional symptoms, conduct problems, hyperactivity/inattention subscales, as well as the total difficulties and impairment scores were used. These subscales were chosen as they are most clinically relevant to the population studied. The SDQ has well-established and excellent psychometric properties [31].

Although SDQ scores are considered as continuous variables, they can be categorised into four bandings. The bandings are: (1) close to average (80% of population); (2) slightly raised (10%); (3) high (5%) and (4) very high (5%). The bandings were created from a large population-based UK survey (https://www.sdqinfo.org).

*Pandemic Anxiety Scale* (PAS; parent self-rating and parent proxy-rating about the CYP): Pandemic-related anxiety was assessed using the 7-item PAS. Items form two subscales representing anxiety about: (1) the disease aspects of COVID-19 (PAS-Disease; 4 items, e.g. fear of catching COVID-19, or fear of transmitting COVID-19) and (2) the consequences of COVID-19 (PAS-Consequence; 3 items, e.g. worries about not having enough food, or worries about future jobs and the economy). Each item is rated on a 5-point scale from “0” (strongly disagree) to “4” (strongly agree), with higher scores indicating greater anxiety. Versions for parent report about their own anxiety and parent report about the child’s anxiety were included in this study. The PAS is being used widely across age groups and languages and early findings suggest good psychometric properties [32].

*Additional items* The Co-space study included a range of additional items and measures (reported elsewhere [33]). Demographic questions included the gender and ethnicity of the CYP and the parent, relationship of the parent-informant to the CYP and employment status of the parent. Other relevant questions included in this study (see “Analysis” section) enquired about access to outside space, time spent outside doing physical activity, and keeping to a similar or regular routine. These questions were created by the co-space study team and response options were categorical.

### Analysis

The analysis compared scores for the three diagnostic groups of interest (CYP with ADHD, ASD and ADHD + ASD) with CYP with no/other mental health disorders (the comparator/reference group). Descriptive statistics [mean (s.d.)] are initially presented for the observed scores at baseline (whole sample) and 1-month follow-up. Changes in the measures from baseline to 1-month follow-up (using multiply imputed datasets) were then compared across the four groups using linear regression models, adjusting for child age, child gender, timing of the questionnaire, pandemic anxiety measured via PAS disease and consequence subscales at baseline, time spent outside doing physical activity, and keeping to a similar or regular routine*.* The analysis adjusted for these variables as the literature indicates that they may be confounding factors [26, 34–38]. Missing data were imputed using multiple imputation by chained equations. The imputation model included the baseline score, child gender, child age and timing of questionnaire.

The assumptions of homoscedasticity and normality were checked using descriptive data and plots, without formal statistical hypothesis testing. Homoscedasticity was checked by informally comparing standard deviation of change scores in each group, while the normality of change scores was inspected using histograms.

The results from the imputed models are presented as differences in means for the change scores over the follow-up period, with the no/other mental health disorder group is the reference group. A positive score in the imputed model means that the average change (between the baseline and follow-up time-points) is greater in the diagnostic group of interest compared with the reference group. To elaborate, a positive score indicates that the diagnostic group of interest has done less well over time (in terms of the difference between baseline and follow-up scores) than the reference group. Conversely, a negative score means that the average change is less in the diagnostic group than in the reference group., i.e. a negative score indicates that the diagnostic group of interest has done better over time (in terms of the difference between baseline and follow-up scores) than the reference group.

In terms of the direction of within-group change (i.e. whether the diagnostic groups of interest improve or deteriorate over the follow-up period), the outputs from the imputed models should be interpreted with reference to the observed mean scores at baseline and follow-up.

## Results

The sample size was 6507 CYP, with a mean age of 9.4 years; 51% were male and 90% were White British/Irish ethnicity. Approximately 2.5% (n = 160) of the sample were reported as having ADHD, 7% ASD (n = 465) and 2.4% (n = 155) both ADHD and ASD, (Table 1). CYP in the diagnostic groups of interest were more likely to be male and slightly younger than those in the reference group. Follow-up data were available for approximately 50% (n = 3114) of the sample; similar characteristics and scores at baseline were observed between those with and without follow-up data (see Appendix 1).

### Strengths and Difficulties Questionnaire (SDQ)

At baseline and follow-up, scores on the SDQ indicate that CYP with ADHD and/or ASD scored higher than the comparison group on all SDQ domains (Table 2). Baseline responses presented in fourfold classification bandings (close to average, slightly raised, high, very high) are presented in Appendix 2. Appendix 2 also contains the completion rates for each scale.

**Table 2:** Baseline and change scores for Strengths and Difficulties Questionnaire by group

	ADHD without ASD (n = 160)	ASD without ADHD (n = 465)	ADHD and ASD (n = 155)	Reference group (n = 5727)	Total (n = 6507)
Emotional symptoms					
Baseline mean [sd] Baseline (n)	4.5 [2.7] (63)	5.5 [2.7] (241)	6.1 [2.6] (72)	2.8 [2.4] (2738)	3.1 [2.6] (3114)
Follow-up mean [sd] Follow-up (n)	4.0 [2.8] (63)	5.2 [2.9] (241)	6.0 [2.9] (72)	2.8 [2.5] (2737)	3.1 [2.7] (3113)
Change mean^a^ [sd] Change (n)	− 0.5 [1.8] (63)	− 0.3 [2] (241)	− 0.1 [1.8] (72)	0 [1.8] (2737)	0 [1.8] (3113)
Difference in means of change score^b^ over 1-month follow-up (95%CI)	− 0.29 (− 0.73, 0.15) p = 0.198	− 0.31 (− 0.53, − 0.09) p = 0.006	− 0.09 (− 0.46, 0.28) p = 0.635	0	–
Conduct problems					
Baseline mean [sd] Baseline (n)	3.7 [2.0] (63)	3.1 [2.1] (241)	4.9 [2.5] (72)	1.7 [1.6] (2738)	1.9 [1.8] (3114)
Follow-up mean [sd] Follow-up (n)	3.8 [2.1] (63)	3.1 [2.1] (241)	4.2 [2.3] (72)	2 [1.7] (2737)	2.1 [1.9] (3113)
Change mean^a^ [sd] Change (n)	0.2 [1.5] (63)	− 0.1 [1.5] (241)	− 0.7 [1.4] (72)	0.2 [1.3] (2737)	0.2 [1.3] (3113)
Difference in means of change score^b^ over 1-month follow-up (95%CI)	− 0.11 (− 0.39, 0.18) 0.476	− 0.23 (− 0.40, − 0.06) 0.008	− 0.75 (− 1.02, − 0.47) < 0.001	0	–
Hyperactivity/inattention					
Baseline mean [sd] Baseline (n)	8.5 [1.7] (63)	6.3 [2.6] (241)	8.9 [1.5] (72)	3.9 [2.6] (2738)	4.3 [2.8] (3114)
Follow-up mean [sd] Follow-up (n)	8.5 [1.9] (63)	6.3 [2.7] (241)	8.6 [1.7] (72)	4.4 [2.6] (2737)	4.7 [2.8] (3113)
Change mean^a^ [sd] Change (n)	0 [1.1] (63)	0 [1.8] (241)	− 0.3 [1.4] (72)	0.5 [1.9] (2737)	0.4 [1.8] (3113)
Difference in means of change score^b^ over 1-month follow-up (95%CI)	− 0.46 (− 0.83, − 0.10) p = 0.013	− 0.45 (− 0.70, − 0.20) p ≤ 0.001	− 0.70 (− 1.04, − 0.36) p ≤ 0.001	0	–
Total symptoms					
Baseline mean [sd] Baseline (n)	20.6 [6] (63)	20.2 [6.1] (241)	25.4 [5.7] (72)	10.1 [6] (2738)	11.5 [7.1] (3114)
Follow-up mean [sd] Follow-up (n)	20.5 [6.6] (63)	20 [6.4] (241)	24.4 [6.6] (72)	11.2 [6.4] (2735)	12.4 [7.2] (3111)
Change mean^a^ [sd] Change (n)	− 0.1 [3.7] (63)	− 0.2 [4] (241)	− 0.9 [3.7] (72)	1 [4] (2735)	0.9 [4.1] (3111)
Difference in means of change score^b^ over 1-month follow-up (95%CI)	− 0.81 (− 1.71, 0.10) p = 0.081	− 1.16 (− 1.64, − 0.68) p ≤ 0.001	− 1.68 (− 2.53, − 0.83) p ≤ 0.001	0	–
Impairment					
Baseline mean [sd] Baseline (n)	4.4 [2.4] (63)	5 [2.7] (240)	6.5 [2.4] (72)	0.7 [1.6] (2737)	1.2 [2.3] (3112)
Follow-up mean [sd] Follow-up (n)	4.9 [2.7] (63)	5.2 [2.9] (239)	6.7 [2.5] (72)	0.9 [1.7] (2732)	1.4 [2.4] (3106)
Change mean^a^ [sd] Change (n)	0.5 [1.8] (63)	0.2 [1.9] (238)	0.2 [1.4] (72)	0.2 [1.2] (2731)	0.2 [1.3] (3104)
Difference in means of change score^b^ over 1-month follow-up (95%CI)	0.36 (0.08, 0.64) p = 0.013	− 0.01 (− 0.16, 0.15) p = 0.930	0.13 (− 0.16, 0.42) p = 0.387	0	–

Reference group contained children and young people with no or other mental health disorders that were not ADHD and/or ASD

*ADHD* attention deficit hyperactivity disorder, *ASD* autism spectrum disorder

^a^The mean within-individual change scores from baseline to 1-month follow-up

^b^Adjusted imputed analyses (n = 6507): The difference in means of change scores from baseline to 1-month follow-up compared with the reference group (other or no mental health disorder)

**Fig. 1:**
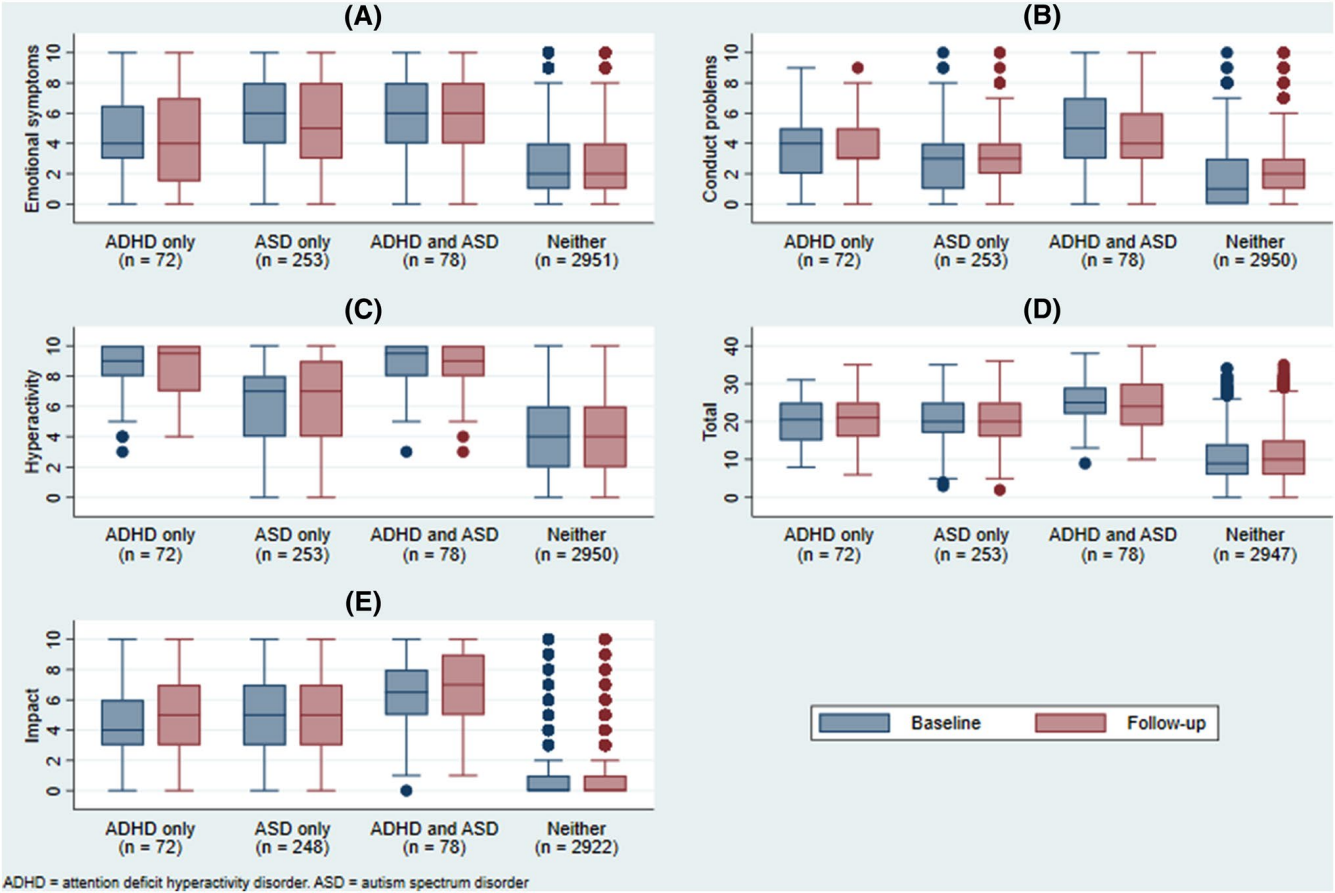
Observed baseline and follow-up scores on SDQ. **A** Emotional symptoms, **B** Conduct problems, **C** Hyperactivity/inattention, **D** Total difficulties, **e** Impairment

Observed baseline and follow-up scores (reflecting complete-case data) are shown in Fig. 1 and suggest a slight reduction in emotional symptoms in the ASD-only group and ADHD + ASD groups. Change mean scores indicate a slight improvement in these diagnostic groups. The imputed analysis demonstrated that there was significantly greater change in the ASD-only group compared to the reference group, reflecting a reduction in emotional symptoms.

For conduct problems, observed scores showed slight increases for the ADHD-only and reference groups, whereas slight reductions were noted for the ASD-only and ADHD + ASD groups. Imputed analysis confirmed that there was a modest reduction in conduct symptoms in these two ASD groups compared to the reference group, where there was a slight increase.

For hyperactivity/inattention, observed scores suggest a slight reduction for the ADHD + ASD group, and an increase for the reference group. Imputed analysis revealed greater changes in scores for the reference group compared with all three diagnostic groups. Similarly, for total symptoms, observed scores suggested a deterioration amongst the reference group. The imputed analysis confirmed that over time, scores reduced more in ASD only and ADHD + ASD groups than in the reference group.

In contrast, for impairment, observed scores suggested a deterioration among all four groups. However, the imputed analysis showed that the ADHD-only group appeared to show a greater reduction in scores over time than the reference group.

It should be noted, that overall, despite a tendency for greater reduction in scores in the diagnostic groups of interest, scores remained higher in these groups than that of the reference group.

### Pandemic Anxiety Scale (PAS)

Table 3 shows that at baseline, parents of CYP in the diagnostic groups reported slightly higher mean scores on the PAS disease sub-scale than the reference group for both their self-ratings and proxy ratings about their child. Across all groups, the self-rated parent observed scores decreased over time. For the proxy ratings, observed scores remained stable over time, except for the ASD-only group which increased.

**Table 3:** Pandemic anxiety of the parent and child proxy scores on the pandemic anxiety scale

	ADHD without ASD (n = 160)	ASD without ADHD (n = 465)	ADHD and ASD (n = 155)	Reference group (n = 5727)	Total (n = 6507)
Parent scores					
Disease subscale					
Baseline mean [sd] Baseline (n)	10.5 [3.4] 63	10.3 [3.3] 240	11.1 [3.4] 71	9.9 [3.1] 2732	9.9 [3.2] 3106
Follow-up mean [sd] Follow-up (n)	9.4 [3.5] (63)	9.6 [3.4] (241)	9.8 [3.8] (72)	8.8 [3.3] (2736)	8.9 [3.4] (3112)
Change mean^a^ [sd] Change (n)	− 1.1 [2.1] (63)	− 0.7 [2.3] (240)	− 1.3 [2.7] (71)	− 1.1 [2.5] (2730)	− 1.1 [2.4] (3104)
Difference in means of change score^b^ over 1-month follow-up (95%CI)	0.14 (− 0.40, 0.68) p = 0.606	0.38 (0.07, 0.69) p = 0.016	− 0.18 (− 0.80, 0.43) p = 0.566	0	–
Consequence subscale					
Baseline mean [sd] Baseline (n)	6.4 [3.6] 63	5.9 [3] 240	6.7 [3] 72	5.8 [2.9] 2735	5.8 [2.9] 3110
Follow-up mean [sd] Follow-up (n)	6.3 [3.2] (63)	5.5 [2.9] (240)	6.1 [2.9] (72)	5.4 [2.9] (2736)	5.4 [2.9] (3111)
Change mean^a^ [sd] Change (n)	− 0.1 [2.1] (63)	− 0.4 [2.4] (239)	− 0.6 [2.4] (72)	− 0.4 [2.2] (2733)	− 0.4 [2.3] (3107)
Difference in means of change score^b^ over 1-month follow-up (95%CI)	0.45 (− 0.09, 0.99) p = 0.102	0.03 (− 0.29, 0.34) p = 0.864	− 0.08 (− 0.60, 0.44) p = 0.760	0	–
Child (proxy) scores					
Disease subscale					
Baseline mean [sd] Baseline (n)	7.6 [3.7] (63)	7.9 [4.3] (241)	8 [4.2] (72)	6.6 [3.3] (273)6	6.7 [3.4] (3112)
Follow-up mean [sd] Follow-up (n)	7.6 [3.7] (63)	8.3 [3.9] (241)	8.1 [4.1] (72)	6.6 [3.4] (2736)	6.8 [3.5] (3112)_
Change mean^a^ [sd] Change (n)	0 [2.7] (63)	0.4 [2.8] (241)	0 [2.9] (72)	0 [2.6] (2734)	0 [2.6] (3110)
Difference in means of change score^b^ over 1-month follow-up (95%CI)	0.08 (− 0.49, 0.65) p = 0.785	0.40 (0.02, 0.78) p = 0.037	− 0.18 (− 0.80, 0.44) p = 0.566	0	–
Consequence subscale					
Baseline mean [sd] Baseline (n)	3.8 [3.1] (63)	3.5 [2.8] (241)	3.3 [2.9] (72)	3.3 [2.4] (2736)	3.3 [2.5] (3112)
Follow-up mean [sd] Follow-up (n)	3.4 [3.1] (63)	3.6 [2.7] (241)	3.5 [2.7] (72)	3.2 [2.5] (2737)	3.2 [2.5] (3113)
Change mean^a^ [sd] Change (n)	− 0.4 [2.6] (63)	0 [2.2] (241)	0.3 [2.1] (72)	− 0.1 [2] (2736)	− 0.1 [2.1] (3112)
Difference in means of change score^b^ over 1-month follow-up (95%CI)	− 0.13 (− 0.64, 0.38) p = 0.623	− 0.02 (− 0.28, 0.24) p = 0.894	0.24 (− 0.23, 0.71) p = 0.318	0	–

Reference group contained children and young people with no or other mental health disorders that were not ADHD and/or ASD

*ADHD* attention deficit hyperactivity disorder, *ASD* autism spectrum disorder

^a^The mean within-individual change scores from baseline to 1-month follow-up

^b^Adjusted imputed analyses (n = 6507): The difference in means of change scores from baseline to 1-month follow-up compared with the reference group (other or no mental health disorder)

Results from the imputed analysis demonstrated that parents of CYP with ASD-only reported greater change in their own anxiety (p = 0.016) and also that of their child (p < 0.037) than the reference group in relation to the disease sub-scale. Observed scores show a greater deterioration for the ASD group.

**Fig. 2:**
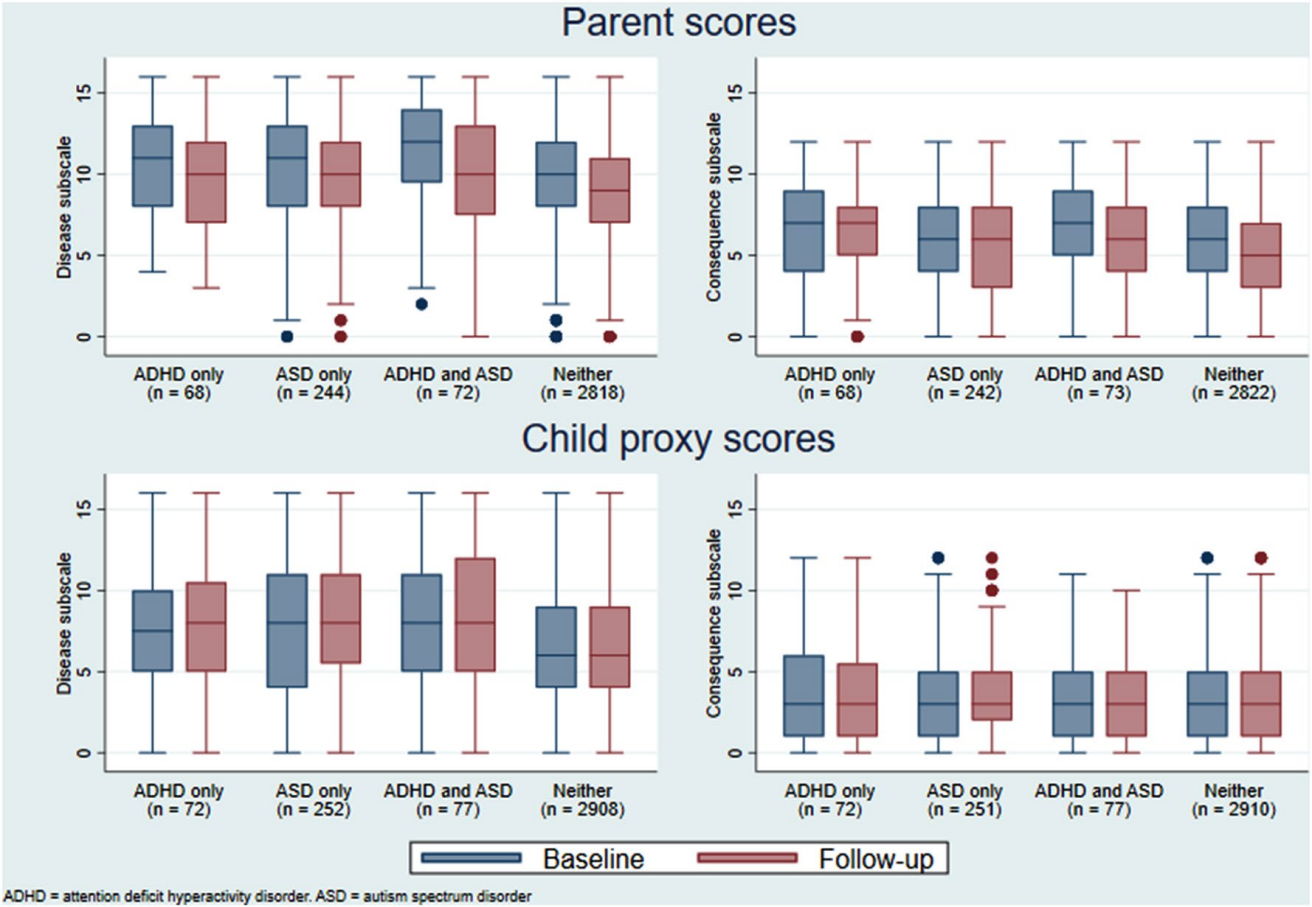
Observed baseline and follow-up parent (top) and child proxy (bottom) scores on disease (left) and consequence (right) Pandemic Anxiety Scale

With reference to the consequence sub-scale of the PAS (Fig. 2), observed scores shows a reduction in parent self-rated anxiety across all groups. Results from the imputed analysis did not reveal any significant differences for either parent self-rated or child-proxy rated scores.

## Discussion

Given the uncertainty about the mental health impacts of the pandemic for potentially vulnerable people with neurodevelopmental disorders such as ADHD or ASD, we investigated outcomes following the onset of the utilising data from the Co-Space longitudinal study [27]. There was a mixed pattern of findings with CYP with ADHD and/or ASD showing greater reduction in symptom scores in some domains but less reduction in others. In terms of pandemic anxiety, our findings suggested that in the initial stages of the pandemic, CYP with ADHD and/or ASD had higher baseline scores than other CYP on the PAS disease sub-scale. At 1-month follow up, there appeared to be an increase in pandemic anxiety for CYP with ASD. However, this deterioration was not reflected in corresponding changes in emotional or behavioural difficulties (SDQ), perhaps reflecting specific worries about disease aspects around catching or transmitting COVID-19. In terms of the SDQ, at 1-month follow up, the change scores indicated that CYP with ADHD and/or ASD tended to either show greater improvements, or less deterioration, in emotional and behavioural difficulties than other CYP. That is, CYP in these diagnostic groups were reported to experience fewer emotional and behavioural difficulties than were present at baseline. However, in terms of functional impairment related to emotional and behavioural difficulties, change scores indicated that all groups appeared to do worse over the follow-up period. In particular, the ADHD group showed increased impairment over time, compared with the reference group, despite improvements or no changes in symptoms. It is noteworthy that despite instances of greater reductions/less deterioration than the reference group, CYP with neurodevelopmental disorders still had higher symptom scores on the SDQ over time.

Despite concerns about the potential vulnerability of CYP with neurodevelopmental disorders, our findings did not indicate a significant worsening of emotional or behavioural symptoms over the initial 1-month period. Although it is difficult to draw definitive comparisons with other studies given the heterogeneity in timings, sample characteristics and analysis, our findings appear in keeping with other population-based studies which suggest little/no deterioration and/or possible improvements in mental health symptoms during the initial pandemic period [15, 39]. Given the lack of condition-specific research, comparisons with existing research are limited, however, our findings support those of Houghton et al. [10] whose longitudinal study in Australia also showed little change in mental health symptoms for CYP with NDD. Our findings contrast with those of Nonweiler et al. [25] who conducted a cross-sectional study and found that CYP with neurodevelopmental disorders showed worsening of emotional and conduct symptoms during COVID compared to pre-COVID. This difference might be explained by our longitudinal design and different analytical approach, adjusting controlled for factors (e.g. routine, activity, time spent outside) which previous research has indicated may be important for CYP with neurodevelopmental disorders [26, 35–38]. It is possible that these factors provide at least partial explanation as to why Bobo et al. [20] found heterogeneity in outcomes for CYP with ADHD during the pandemic, with one-third showing improvement, one-third stability, and one-third symptom decline.

The findings that CYP with ADHD and/or ASD showed some small improvements in emotional and behavioural difficulties compared with the reference group may reflect possible differential impact between the groups of lockdown and school closures. It has been proposed that school closures may reduce academic and social pressures for children and young people [40], particularly for those who may struggle in classroom and school environments. However, the increase in impairment for CYP with ADHD might reflect the negative aspects of home confinement with limited access to outdoor space, friendship groups and usual activities.

In terms of study strengths, this study is amongst the first to present longitudinal data explore COVID-19 impact over time. The large sample size strengthens the study findings. The study also combined both validated and novel pandemic-specific measures to explore changes on established measures and COVID-19 changes. The study is also unique in controlling for a range of important confounders (such as routine changes and activity). However, there are also certain study limitations with the data that are important to recognise to inform cautious interpretation of the results. The sample were not a representative sample as they were self-selected, predominately white, British, employed and the responders were typically female parents, limiting generalisability of the findings. We do not have pre-pandemic data for the sample so baseline scores may reflect both pre-pandemic and in-pandemic difficulties. We therefore use our two time-points (baseline and 1-month follow-up) as an insight into short-term longitudinal changes in the early stages of the pandemic. As participants could sign-up to the Co-Space study over a given time period, the baseline and 1-month follow-up were not at exactly the same point in time for all participants. To mitigate this, we adjusted for questionnaire timing in the analysis. Additionally, we relied on parent-reported information and there was considerable attrition over time, although there was no evidence of selection bias in terms of follow-up responders. The primary analysis relied upon multiple imputation of missing data. Because of the limitations of multiple imputation in the presence of considerable attrition, guidelines recommend that these findings should be considered as hypothesis generating [41]. We also acknowledge that our sample size for the disorder groups (particularly the ADHD groups) were small. Although parents are important and reliable informants about their child’s mental health [42] the absence of child-completed measures is a limitation of our study. We also acknowledge that we relied on parent reports of clinical diagnosis, which were not validated by a clinician report. Given the scale and design (large online national survey) of this study, it was not possible to do this, however, parental reports have been shown to predict independent clinical diagnosis, indicating the validity of our approach [43]. Finally, a large number of comparisons were performed which may have increased the risk of chance findings; we therefore also presented observed change scores to aid interpretation of the results. Although our findings should be interpreted cautiously, they are useful to guide future research. This is important as there is a lack of large-scale pandemic-related research focusing on CYP with ADHD and/or ASD.

In terms of clinical and service implications, the findings from this longitudinal study highlight that CYP with neurodevelopmental disorders showed some small improvements in emotional and behavioural symptoms during the initial period of the pandemic, despite enduring levels of pandemic disease anxiety. Despite this small improvement, scores for emotional and behavioural symptoms remained higher for CYP with a neurodevelopmental disorder compared to those without. There was also an increase in functional impairment among CYP with ADHD. Given the exploratory nature of our study, these findings should be interpreted with caution and further research is required to fully understand the longer-term impact of the pandemic. As the possibility of further lockdowns or school closures remains, it is important for key adults such as parents, teachers, and clinicians to be aware of these findings while keeping in mind the importance of both individual and group-level vulnerabilities. It is also crucial that practitioners continue to remain mindful of, and sensitive to, the possible longer-term impacts of pandemic-related confinements and subsequent expectations around re-adjustments to pre-pandemic routines; where appropriate, asking CYP about their perceptions of these experiences.

## Summary

This study investigated differences in mental health and pandemic anxiety during the first UK lockdown for the COVID-19 pandemic, for CYP with and without ADHD and/or ASD. Baseline and 1-month follow-up data were analysed from the Co-Space study, a UK web-based longitudinal survey investigating the impact of the pandemic for 4–16-year-olds. The measures included the parent-completed questionnaires [Strengths and Difficulties Questionnaire (SDQ) & Pandemic Anxiety Scale (PAS)]. The study found that CYP with ADHD and/or ASD had higher scores on the measures at baseline than CYP without ADHD/ASD. Over the 1-month follow-up period, in general CYP with ASD showed a small but significant improvement in symptoms (SDQ), compared with CYP without ADHD/ASD. Despite this improvement, SDQ scores were still higher. CYP with ASD also reported to have experienced a worsening of disease anxiety (PAS) and CYP with ADHD a deterioration in terms of functional impairment. Although these findings should be interpreted tentatively, they highlight a mixed pattern of pandemic-related impact, with CYP with ADHD/ASD faring better in some domains but worse in others. Clinically, these findings highlight considerable heterogeneity and the need for practitioners to be mindful of individual, rather than group-level, vulnerabilities. Improved awareness and understanding of this heterogeneity in vulnerabilities is important for future pandemic preparedness, particularly in the event of possible further lockdowns or school closures.

## Appendix 1

See Tables 4

**Table 4:** Characteristics of sample by follow-up status

	Follow-up data available (n = 3114)	Follow-up data not available (n = 3393)	Total
Child age			
Mean [sd]	9.1 [3.4]	9.7 [3.6]	9.4 [3.5]
n	3114	3393	6507
Child gender			
Male	1595 (51%)	1737 (51%)	3332 (51%)
Female	1494 (48%)	1635 (48%)	3129 (48%)
Other/prefer not to say	25 (1%)	21 (1%)	46 (1%)
Child ethnicity			
White: British, Irish, other	2879 (92%)	3005 (89%)	5884 (90%)
Mixed race: White/Black & mixed race other	159 (5%)	187 (6%)	346 (5%)
Asian/British	23 (1%)	83 (2%)	106 (2%)
Black/British	10 (< 1%)	20 (1%)	30 (< 1%)
Other/prefer not to say	43 (1%)	98 (3%)	141 (2%)
Relationship of parent/guardian to child			
Parent	3073 (99%)	3277 (97%)	6350 (98%)
Step-parent	11 (< 1%)	35 (1%)	46 (1%)
Grandparent	17 (1%)	34 (1%)	51 (1%)
Other	13 (< 1%)	47 (1%)	60 (1%)
Gender of parent/guardian			
Male	180 (6%)	276 (8%)	456 (7%)
Female	2919 (94%)	3098 (91%)	6017 (92%)
Other/prefer not to say	15 (< 1%)	19 (1%)	34 (1%)
Ethnicity of parent/guardian			
White: British, Irish, other	2978 (96%)	3106 (92%)	6084 (93%)
Mixed race: White/Black & mixed race other	54 (2%)	76 (2%)	130 (2%)
Asian/British	31 (1%)	88 (3%)	119 (2%)
Black/British	9 (< 1%)	23 (1%)	32 (< 1%)
Other/prefer not to say	42 (1%)	100 (3%)	142 (2%)
Employment status of parent/guardian			
In education	45 (1%)	84 (2%)	129 (2%)
Employed	2641 (85%)	2822 (83%)	5463 (84%)
Unable due to disability	62 (2%)	95 (3%)	157 (2%)
Homemaker/full-time parent	325 (10%)	327 (10%)	652 (10%)
Unemployed and seeking work	27 (1%)	54 (2%)	81 (1%)
Retired	14 (< 1%)	11 (< 1%)	25 (< 1%)
Child has special education needs			
No	2608 (84%)	2788 (82%)	5396 (83%)
Yes	506 (16%)	605 (18%)	1111 (17%)
Access to outside space			
No	96 (3%)	180 (5%)	276 (4%)
Yes	3016 (97%)	3211 (95%)	6227 (96%)
Missing	2	2	4
SDQ—emotional symptoms			
Mean [sd]	3.1 [2.6]	3 [2.6]	3.1 [2.6]
n	3114	3372	6486
Missing	0	21	21
SDQ—conduct problems			
Mean [sd]	1.9 [1.8]	2.1 [1.9]	2 [1.9]
n	3114	3371	6485
Missing	0	22	22
SDQ—hyperactivity/inattention			
Mean [sd]	4.3 [2.8]	4.4 [2.8]	4.4 [2.8]
n	3114	3371	6485
Missing	0	22	22
SDQ—total symptoms			
Mean [sd]	11.5 [7.1]	11.8 [7.1]	11.6 [7.1]
n	3114	3366	6480
Missing	0	27	27
SDQ—impact			
Mean [sd]	1.2 [2.3]	1.3 [2.4]	1.3 [2.3]
n	3112	3315	6427
Missing	2	78	80
PAS—disease subscale			
Mean [sd]	9.9 [3.2]	10.1 [3.2]	10 [3.2]
n	3106	2954	6060
Missing	8	439	447
PAS—consequence subscale			
Mean [sd]	5.8 [2.9]	6 [3]	5.9 [2.9]
n	3110	2955	6065
Missing	4	438	442

*ADHD* attention deficit hyperactivity disorder, *ASD* autism spectrum disorder, *SDQ* strengths and difficulties questionnaire, *PAS* pandemic anxiety scale

## Appendix 2

See Tables 5, 6.

**Table 5:** Strengths and difficulties questionnaire at baseline in categorical response group

	ADHD and without ASD	ASD and without ADHD	ADHD and ASD	Reference group	Total
Emotional symptoms					
Mean [sd]	4.5 [2.7]	5.6 [2.6]	5.7 [2.5]	2.8 [2.4]	3.1 [2.6]
Median [25th, 75th centile]	4 [3, 7]	6 [4, 8]	6 [4, 8]	2 [1, 4]	3 [1, 5]
Min, max	0, 10	0, 10	0, 10	0, 10	0, 10
n	160	463	155	5708	6486
Close to average	60 (38%)	109 (24%)	31 (20%)	3859 (68%)	4059 (63%)
Slightly raised	23 (14%)	46 (10%)	16 (10%)	592 (10%)	677 (10%)
High	33 (21%)	120 (26%)	40 (26%)	733 (13%)	926 (14%)
Very high	44 (28%)	188 (41%)	68 (44%)	524 (9%)	824 (13%)
Missing	0	2	0	19	21
Conduct problems					
Mean [sd]	4 [2.2]	3.3 [2.2]	4.7 [2.3]	1.8 [1.7]	2 [1.9]
Median [25th, 75th centile]	4 [2, 5]	3 [2, 5]	4 [3, 6]	1 [0, 3]	2 [1, 3]
Min, max	0, 10	0, 10	0, 10	0, 10	0, 10
n	160	464	155	5706	6485
Close to average	48 (30%)	181 (39%)	27 (17%)	4129 (72%)	4385 (68%)
Slightly raised	21 (13%)	85 (18%)	23 (15%)	756 (13%)	885 (14%)
High	57 (36%)	124 (27%)	54 (35%)	649 (11%)	884 (14%)
Very high	34 (21%)	74 (16%)	51 (33%)	172 (3%)	331 (5%)
Missing	0	1	0	21	22
Hyperactivity/inattention					
Mean [sd]	8.5 [1.6]	6.6 [2.4]	8.8 [1.6]	3.9 [2.6]	4.4 [2.8]
Median [25th, 75th centile]	9 [8, 10]	7 [5, 8]	9 [8, 10]	4 [2, 6]	4 [2, 6]
Min, max	3, 10	0, 10	2, 10	0, 10	0, 10
n	160	464	155	5706	6485
Close to average	11 (7%)	149 (32%)	8 (5%)	4247 (74%)	4415 (68%)
Slightly raised	22 (14%)	120 (26%)	22 (14%)	829 (15%)	993 (15%)
High	30 (19%)	80 (17%)	16 (10%)	270 (5%)	396 (6%)
Very high	97 (61%)	115 (25%)	109 (70%)	360 (6%)	681 (11%)
Missing	0	1	0	21	22
Total symptoms					
Mean [sd]	20.6 [6]	20.7 [6]	24.5 [5.4]	10.3 [6.1]	11.6 [7.1]
Median [25th, 75th centile]	21 [16, 25]	21 [17, 25]	25 [21, 28]	9 [6, 14]	10 [6, 16]
Min, max	7, 35	3, 35	9, 38	0, 36	0, 38
n	160	463	155	5702	6480
Close to average	20 (13%)	52 (11%)	4 (3%)	4187 (73%)	4263 (66%)
Slightly raised	23 (14%)	60 (13%)	8 (5%)	594 (10%)	685 (11%)
High	23 (14%)	67 (14%)	22 (14%)	407 (7%)	519 (8%)
Very high	94 (59%)	284 (61%)	121 (78%)	514 (9%)	1013 (16%)
Missing	0	2	0	25	27
Impact					
Mean [sd]	4.4 [2.5]	5.2 [2.7]	5.9 [2.5]	.8 [1.7]	1.3 [2.3]
Median [25th, 75th centile]	4 [3, 6]	5 [3, 7]	6 [4, 8]	0 [0, 1]	0 [0, 2]
Min, max	0, 10	0, 10	0, 10	0, 10	0, 10
n	159	459	153	5656	6427
Close to average	10 (6%)	22 (5%)	3 (2%)	4134 (73%)	4169 (65%)
Slightly raised	16 (10%)	29 (6%)	5 (3%)	522 (9%)	572 (9%)
High	13 (8%)	27 (6%)	9 (6%)	326 (6%)	375 (6%)
Very high	120 (75%)	381 (83%)	136 (89%)	674 (12%)	1311 (20%)
Missing	1	6	2	71	80

Reference group consisted of children and young people with no or other mental health disorders which were not ADHD and/or ASD

*ADHD* attention deficit hyperactivity disorder, *ASD* autism spectrum disorder

**Table 6:** Data completeness for questionnaires by group

	ADHD without ASD (n = 160)	ASD without ADHD (n = 465)	ADHD and ASD (n = 155)	Reference group (n = 5727)	Total (n = 6507)
Strength and difficulties questionnaire					
Emotional symptoms					
Baseline, n (%)	160 (100%)	463 (100%)	155 (100%)	5708 (100%)	6486 (100%)
Follow-up, n (%)	72 (45%)	253 (54%)	78 (50%)	2951 (52%)	3354 (52%)
Conduct problems					
Baseline, n (%)	160 (100%)	464 (100%)	155 (100%)	5706 (100%)	6485 (100%)
Follow-up, n (%)	72 (45%)	253 (54%)	78 (50%)	2951 (52%)	3354 (52%)
Hyperactivity/inattention					
Baseline, n (%)	160 (100%)	464 (100%)	155 (100%)	5706 (100%)	6485 (100%)
Follow-up, n (%)	72 (45%)	253 (54%)	78 (50%)	2950 (52%)	3353 (52%)
Total symptoms					
Baseline, n (%)	160 (100%)	463 (100%)	155 (100%)	5702 (100%)	6480 (100%)
Follow-up, n (%)	72 (45%)	253 (54%)	78 (50%)	2948 (51%)	3351 (51%)
Impairment					
Baseline, n (%)	159 (99%)	459 (99%)	153 (99%)	5656 (99%)	6427 (99%)
Follow-up, n (%)	72 (45%)	249 (54%)	78 (50%)	2923 (51%)	3322 (51%)
Pandemic anxiety					
Parent scores					
Disease subscale					
Baseline, n (%)	152 (95%)	442 (95%)	143 (92%)	5323 (93%)	6060 (93%)
Follow-up, n (%)	68 (43%)	245 (53%)	73 (47%)	2825 (49%)	3211 (49%)
Consequence subscale					
Baseline, n (%)	152 (95%)	439 (94%)	144 (93%)	5330 (93%)	6065 (93%)
Follow-up, n (%)	68 (43%)	243 (52%)	73 (47%)	2825 (49%)	3209 (49%)
Child (proxy) scores					
Disease subscale					
Baseline, n (%)	157 (98%)	458 (98%)	151 (97%)	5576 (97%)	6342 (97%)
Follow-up, n (%)	72 (45%)	252 (54%)	77 (50%)	2910 (51%)	3311 (51%)
Consequence subscale					
Baseline, n (%)	157 (98%)	460 (99%)	151 (97%)	5576 (97%)	6344 (97%)
Follow-up, n (%)	72 (45%)	251 (54%)	77 (50%)	2911 (51%)	3311 (51%)

Reference group contained children and young people with no or other mental health disorders that were not ADHD and/or ASD

*ADHD* attention deficit hyperactivity disorder, *ASD* autism spectrum disorder
